# Individual-Level Determinants of Lifestyle Behavioral Changes during COVID-19 Lockdown in the United States: Results of an Online Survey

**DOI:** 10.3390/ijerph18084364

**Published:** 2021-04-20

**Authors:** Xiaotao Zhang, Abiodun Oluyomi, LeChauncy Woodard, Syed Ahsan Raza, Maral Adel Fahmideh, Ola El-Mubasher, Jinyoung Byun, Younghun Han, Christopher I. Amos, Hoda Badr

**Affiliations:** 1 Department of Medicine, Section of Epidemiology and Population Science, Baylor College of Medicine, Houston, TX 77030, USA; Xiaotao.Zhang@bcm.edu (X.Z.); Abiodun.Oluyomi@bcm.edu (A.O.); Syed.Raza@bcm.edu (S.A.R.); Maral.Adel@bcm.edu (M.A.F.); Ola.El-Mubasher@bcm.edu (O.E.-M.); chris.amos@bcm.edu (C.I.A.); 2 Department of Health Systems and Population Health Science, University of Houston College of Medicine, Houston, TX 77004, USA; lwoodard@Central.UH.EDU; 3 Humana Integrated Health System Sciences Institute, University of Houston, Houston, TX 77030, USA; 4 Center for Innovations in Quality, Effectiveness, and Safety (IQuESt), Houston, TX 77030, USA; 5 Center for Epidemiology and Population Health, Department of Pediatrics, Baylor College of Medicine, Houston, TX 77030, USA; 6 Institute for Clinical and Translational Research, Baylor College of Medicine, Houston, TX 77030, USA; Jinyoung.Byun@bcm.edu (J.B.); Younghun.Han@bcm.edu (Y.H.)

**Keywords:** COVID-19, coronavirus, lifestyle, anxiety, behavioral determinants

## Abstract

This study examined individual-level determinants of self-reported changes in healthy (diet and physical activity) and addictive (alcohol use, smoking, and vaping) lifestyle behaviors during the initial COVID-19 lockdown period in the USA. A national online survey was administered between May and June 2020 that targeted a representative U.S. sample and yielded data from 1276 respondents, including 58% male and 50% racial/ethnic minorities. We used univariate and multivariable linear regression models to examine the associations of sociodemographic, mental health, and behavioral determinants with self-reported changes in lifestyle behaviors. Some study participants reported increases in healthy lifestyle behaviors since the pandemic (i.e., 36% increased healthy eating behaviors, and 33% increased physical activity). However, they also reported increases in addictive lifestyle behaviors including alcohol use (40%), tobacco use (41%), and vaping (46%). With regard to individual-level determinants, individuals who reported adhering to social distancing guidelines were also more likely to report increases in healthy lifestyle behaviors (β = 0.12, 95% CI 0.04 to 0.21). Conversely, women (β = −0.37, 95% CI −0.62 to −0.12), and unemployed individuals (β = −0.33, 95% CI −0.64 to −0.02) were less likely to report increases in healthy lifestyle behaviors. In addition, individuals reporting anxiety were more likely to report increases in addictive behaviors (β = 0.26, 95% CI 0.09 to 0.43). Taken together, these findings suggest that women and unemployed individuals may benefit from interventions targeting diet and physical activity, and that individuals reporting anxiety may benefit from interventions targeting smoking and alcohol cessation to address lifestyle changes during the pandemic.

## 1. Introduction

The coronavirus disease 2019 (COVID-19) pandemic is an unprecedented public health crisis [1]. Faced with an exponential rise in cases and deaths, and in an effort to avoid overwhelming health systems, countries around the globe have adopted protective measures to mitigate the spread of infection including social distancing and stay-at-home policies [2]. Although such interventions are necessary to mitigate transmission, they may also modify lifestyle behaviors such as diet, physical activity (PA), smoking, and alcohol use [3,4], and have significant consequences for physical health [5].

Healthy lifestyle behaviors such as observing a healthy diet and engaging in recommended amounts of PA are consistently associated with reduced all-cause mortality, and improved health and well-being [6,7]. Increased time spent at home due to adherence with mitigation policies could present an opportunity to practice healthy lifestyle behavior (e.g., by home cooking of healthy meals and engaging in regular PA). Supporting this idea, research has documented increases in population-level interest in PA during the pandemic, although actual change in PA was not ascertained [8]. Simultaneously, increased unstructured time spent at home could contribute to people feeling lonely and distressed, eating when not hungry (i.e., emotional eating), and weight gain [9]. New telework arrangements, temporary closure of fitness facilities, and the need to stay at home could also force abrupt changes in PA, increasing the likelihood of sedentary behavior and weight gain [10,11].

Addictive lifestyle behaviors such as smoking and alcohol use are major contributors to the global burden of disease [12] and are associated with poor health outcomes [13]. Emerging evidence has suggested that slight increases in these behaviors during the pandemic [14,15,16] could possibly be in response to the stress, boredom, and social isolation caused by COVID-19 mitigation strategies [17,18,19]. In fact, a market research survey conducted by Nielsen in March 2020, during the initial lockdown period in the USA, showed that alcohol sales increased by 55% in a single week [20]. This is alarming because research has shown that individuals who started abusing substances during the SARS pandemic experienced persistent substance abuse that lasted well beyond the pandemic [21]. Meanwhile, a recent cross-sectional study of 336 U.S. adults found that 28.3% reduced tobacco smoking and 24.9% reduced vaping (e-cigarettes) during the pandemic [22].

Overall, the observed changes in lifestyle behaviors during the pandemic suggest that different people have been affected in different ways. Specifically, more leisure time at home and the threat of becoming severely ill with COVID-19 may have motivated some people to engage in more healthy lifestyle behaviors, and increased distress and changes in social patterns may have driven other people to self-medicate through substance use and abuse [23,24]. Previous studies that have focused on lifestyle behaviors during the pandemic have been largely conducted in Europe and Asia [3,11,25] and with only eating and physical activity [10,26] or smoking and drinking [22,27] as the main outcomes.

In this population-based study, we examined individual-level determinants of self-reported changes in healthy (i.e., diet and physical activity) and addictive (i.e., alcohol use, smoking, and vaping) lifestyle behaviors during the initial COVID-19 lockdown period that was observed by 42 out of 50 states in the USA. Given that healthy and addictive behaviors are not mutually exclusive, we opted to take a more comprehensive approach than previous studies and assessed both healthy behavioral changes (eating and physical activity) and addictive behavioral changes (smoking, vaping, and alcohol drinking). This study also extends the existing knowledge base by evaluating individual-level determinants of lifestyle behavioral changes during the pandemic, including sociodemographic, mental health, and behavioral (e.g., adherence to mitigation strategies) factors. By elucidating individual-level determinants, at-risk groups can be identified, and targeted lifestyle interventions can be developed.

## 2. Materials and Methods

### 2.1. Sample and Setting

This study was a national, population-based survey and it was approved by the Baylor College of Medicine Institutional Review Board (H-47505) and reports on baseline data were obtained from an ongoing longitudinal cohort study of the psychosocial and health behavioral impacts of the pandemic [28]. Eligible individuals were aged ≥18 and resided in the USA. Surveys were distributed in English and Spanish via paid and unpaid social media advertisements and an online survey crowdsourcing platform, Soapbox Sample, during the initial lockdown period in the USA. The survey was initially launched on 13 April 2020 and continued through to 8 June 2020. This period of time coincided with the initial lockdown period that was observed in 42 of 50 states [29]. The U.S. states that did not institute stay-at home orders were Arkansas, Iowa, Nebraska, North Dakota, Oklahoma, South Dakota, Utah, and Wyoming. By the end of the first week in June, rates of infection in the USA had begun to slowly decline, stay-at-home orders in most states had expired, and 34 states had either reopened or were in the process of a phased, state-wide reopening [29]. In addition, governors in 8 hard-hit states had allowed counties or regions that met criteria for slowing the outbreak to open (California, Illinois, Michigan, New York, Oregon, Pennsylvania, Tennessee, and Washington) [29].

Two weeks after the initial survey launch (1 May 2020), the survey was amended to also include questions about lifestyle behaviors, as these questions were not included in the original survey. Consequently, the total survey sample size was 2222, but only 1276 individuals took the survey between 1 May 2020 and 8 June 2020, and therefore comprise the current study sample.

### 2.2. Procedures

Social media advertisements contained a hyperlink directing individuals to the survey website. The landing page contained a brief cover letter describing the study. If, after reading the letter, individuals were interested in participating, they were asked to check a box confirming their eligibility, understanding, and consent. The survey was administered on the Qualtrics survey platform (Provo, UT, USA) [30]. Detailed measures were shown in Appendix A-Table A1 Measures

#### 2.2.1. Lifestyle Behavioral Change Variables

Self-reported changes in healthy (i.e., eating healthy foods and PA) and addictive (i.e., alcohol use, tobacco smoking, and vaping) lifestyle behaviors were assessed and are described as follows:

Healthy Eating. Respondents indicated a degree of agreement with the question, “Since COVID-19, I am eating more healthy foods”. Response options were on a 5-point Likert-type scale from 1 = “strongly disagree” to 5 = “strongly agree”.

Physical Activity. PA was assessed with the item, “Since COVID-19, I am exercising more.” Response options were on a 5-point Likert-type scale from 1 = “strongly disagree” to 5 = “strongly agree”.

Alcohol Use. Individuals were first asked if they drink alcohol (yes/no), and if so, whether their alcohol consumption had “increased”, “decreased”, or “stayed the same” since the pandemic.

Tobacco Smoking. Items were taken from the Global Adult Tobacco Survey [31]. Individuals were first asked if they currently smoke tobacco “on a daily basis”, “less than daily”, or “not at all”. Then, they were asked if their smoking had “increased”, “decreased”, or “stayed the same” since the pandemic.

Vaping. Participants were first asked if they use e-cigarettes “on a daily basis”, “less than daily”, or “not at all”. Then, they were asked if their vaping had “increased”, “decreased”, or “stayed the same” since the pandemic.

Lifestyle behavioral change indices.

Healthy lifestyle behavior change index. The two healthy lifestyle behavioral change variables (i.e., healthy eating and PA) were re-coded by assigning a value of +1 for affirmative responses (i.e., agree or strongly agree), −1 for negative responses (i.e., disagree or strongly disagree), and 0 for neutral responses. Then, scores for the re-coded variables were summed to yield a healthy behavioral change index with a range from −2 to +2. 

Addictive lifestyle behavior index. The three addictive lifestyle behavioral change variables (i.e., alcohol use, tobacco smoking, and vaping) were re-coded as −1 = decrease in behavior, 0 = no change, or +1 = increase in behavior. Then, scores for the re-coded variables were summed to yield an addictive behavioral change index with a range from −3 to +3.

#### 2.2.2. Individual-Level Determinants

Sociodemographic, mental health, and behavioral (i.e., degree of adherence to COVID-19 mitigation strategies) determinants were assessed.

##### Sociodemographics

Individuals were asked about their age, gender, race/ethnicity, education, marital status, annual household income, work status, current living arrangement (alone, or with a spouse/partner, family member, or non-family member), number of household residents, and whether they lived with someone over age 65 or younger than age 18. We also asked individuals about their postal zip codes and cross-streets. On the basis of this information, the states of residence were divided into one of 4 major U.S. census regions, i.e., Northeast, South, Midwest, and West.

##### Mental Health

Mental health over the past 7 days was assessed using the 4-item short-form Patient-Reported Outcome Measure Information System (PROMIS) depression [32] and anxiety measures [33]. For both measures, responses range from 1 (never) to 5 (always) and are summed to form a raw score that can then be scaled into a T-score (standardized) with a mean of 50.0 and standard deviation of 10.0. Scores >60.0 indicate the need for further psychological evaluation [34].

##### Behavioral Determinants 

Self-reported adherence to three COVID-19 mitigation strategies (stay-at-home orders, social distancing, and hand hygiene/sanitization) were assessed. With regard to stay-at-home orders, we first asked, “Is the area where you live currently under a ‘Stay-at-Home’, ‘Safer-at-Home’, or ‘Shelter-at-Home’ order? (yes/no)” If participants responded, “yes”, we then asked, “To what extent do you currently follow the stay-at-home order?” Response options were on an 11-point Likert-type scale from 0 = “not following the order at all” to 10 = “completely following the order”. With regard to social distancing, we asked, “What amount of social distancing do you currently practice?” Response options were on an 11-point Likert-type scale from 0 = “no social distancing at all” to 10 = “complete social distancing”. Finally, to assess hand hygiene/sanitization, we asked, “How often do you practice protective measures like hand washing, use of hand sanitizer, or disinfection of household surfaces to keep yourself and others you live with from contracting COVID-19?” Response options were on an 11-point Likert-type scale from 0 = “never” to 10 = “every few hours”.

### 2.3. Statistical Analysis

Descriptive statistics for all the variables were calculated including the mean, standard deviation (SD), median, and range for continuous variables and relative frequency for categorical variables. For the main study analyses, Pearson’s chi-square (χ^2^) and one-way analysis of variance (ANOVA) were first used to assess univariate associations between each of the individual-level determinant variables and each of the individual lifestyle behavioral change variables (i.e., healthy eating, PA, tobacco smoking, alcohol use, and vaping). Next, univariate regression analyses were conducted to examine associations between each of the individual-level determinant variables and the two lifestyle behavioral change indices (i.e., healthy and addictive behavioral change). Then, all the variables that were associated with the behavioral change indices from the univariate regression analyses (*p* < 0.10) were entered into separate multivariable linear regression models. All statistical analyses were performed in SAS V.9.4 (SAS Institute Inc., Cary, NC, USA).

## 3. Results

### 3.1. Sample Characteristics

Data were derived from 1276 survey respondents. As shown in Table 1, the study sample was predominantly male (58%), middle aged (mean = 45.0 years, SD = 17.0 years), and college educated (79%). Half identified as racial/ethnic minorities and 51% were married. For mental health, the mean of the PROMIS depression T-score was 58.9 (SE = 10.6), which is significantly higher than the U.S. population norm (mean = 50.0, SD = 10.0, *p* < 0.0001). Thirty-nine percent of survey respondents scored above the PROMIS threshold for depression. The mean of the PROMIS anxiety T-score was 56.1 (SE = 10.1), which is also significantly higher than the U.S. population norm (mean = 50.0, SD = 10, *p* < 0.0001). About 48% of survey respondents scored above the PROMIS threshold for anxiety. Approximately 90% of survey respondents lived in an area that was under a stay-at-home order but only 34%, 32%, and 35% reported complete adherence (10 on a scale of 0 = not at all to 10 = completely) to stay-at-home, social distancing, and personal protective behavioral guidelines, respectively. With regard to healthy lifestyle behaviors, 36% of survey respondents agreed or strongly agreed that they were eating more healthy foods and 33% agreed or strongly agreed that they were exercising more since the start of the pandemic. For unhealthy lifestyle behaviors, 40% of survey respondents reported increased alcohol use, 41% reported increased tobacco smoking, and 46% reported increased vaping since the start of the pandemic.

### 3.2. Changes in Healthy Lifestyle Behaviors

#### 3.2.1. Univariate Analyses: Healthy Lifestyle Behavioral Change

Detailed results of Pearson’s chi-square (χ^2^) and one-way ANOVAs to assess associations between each of the individual-level determinant variables and each of the healthy lifestyle behavioral change variables are presented in Supplemental Table S1.

#### 3.2.2. Univariate Analyses: Healthy Behavioral Change Index

As Table 2 shows, older age, female gender, living with someone aged >65, and unemployed work status were all negatively associated with self-reported changes in healthy lifestyle behaviors. Conversely, Black and Hispanic race/ethnicity, being college educated, married, having a household income over USD 75,000, living with someone aged <18, being more adherent to stay-at-home and social distancing guidelines, and practicing more hand hygiene/sanitization were all positively associated with increases in healthy lifestyle behaviors.

#### 3.2.3. Multivariable Analysis: Healthy Behavioral Change Index

**Figure 1:**
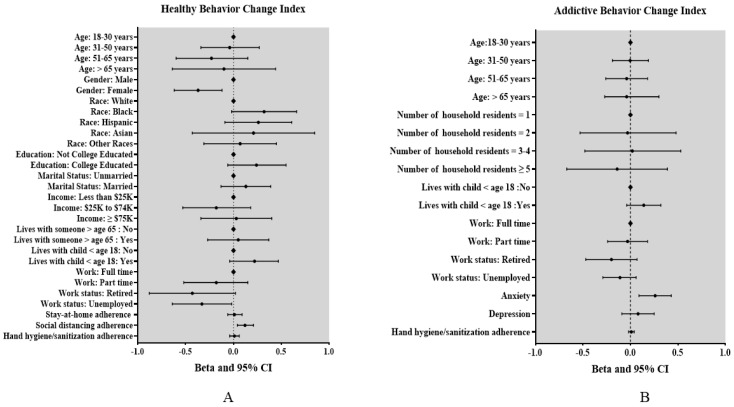
(**A**) Multivariable linear regression model estimating associations between explanatory variables and healthy behavioral change index. Vertical line represents null of beta. Bars denote 95% confidence interval. Healthy lifestyle behaviors include healthy eating and physical activity. Social distancing, stay-at-home adherence, and hand hygiene/sanitization adherence are in one unit increase; (**B**) multivariable linear regression model estimating associations between explanatory variables and addictive behavioral change index. Vertical line represents null of beta. Bars denote 95% confidence interval. Addictive behaviors include smoking, vaping, and drinking. Social distancing, stay-at-home adherence and hand hygiene/sanitization adherence are in one unit increase. ◆ indicate the reference groups.

Multivariable regression revealed that individuals who adhered more to social distancing guidelines were more likely to engage in more healthy lifestyle behaviors (β = 0.12, 95% CI 0.04 to 0.21), relative to those who adhered less In addition, women (β = −0.37, 95% CI −0.62 to −0.12), and unemployed individuals (β = −0.33, 95% CI −0.64 to −0.02) were less likely to report engaging in more healthy lifestyle behaviors, relative to men and employed individuals (Figure 1A and Table 2).

### 3.3. Changes in Addictive Lifestyle Behaviors

#### 3.3.1. Univariate Analyses: Addictive Lifestyle Behavioral Change

Detailed results of the Pearson’s chi-square (χ^2^) and one-way ANOVAs to assess associations between each of the individual-level determinants and each of the addictive lifestyle behavioral change variables are presented in Supplemental Table S2.

#### 3.3.2. Univariate Analyses: Addictive Behavioral Change Index

As Table 3 shows, older aged and retired individuals were less likely to report increases in addictive behaviors, whereas individuals who lived in larger households, with someone aged <18, had anxiety or depression, were less likely to practice hand hygiene/sanitization, and were more likely to report increased addictive behaviors.

#### 3.3.3. Multivariable Analysis: Addictive Behavioral Change Index

Multivariable liner regression analyses revealed that individuals who had anxiety were more likely to report increases in addictive behaviors since the start of the pandemic relative those who did not have anxiety (β = 0.26, 95% CI 0.09 to 0.43) (Figure 1B and Table 3).

## 4. Discussion

This study examined self-reported changes in healthy and addictive lifestyle behaviors during the initial COVID-19 lockdown period in the USA. Consistent with previously published pandemic-focused research [14,15,16], we detected a significant increase in addictive behaviors during lockdown. Nearly two in five people who smoked tobacco or drank alcohol reported increases in these behaviors and, one in two people who vaped reported increased vaping behavior. We also found a significant, albeit a smaller increase in healthy lifestyle behaviors, with about one in three people reporting more healthy eating and PA.

Overall, this study adds to the body of work on lifestyle behavioral changes during the COVID-19 pandemic. We identified sociodemographic, mental health, and behavioral determinants of behavioral changes. Specifically, we found that individuals who practiced more social distancing reported increased healthy behaviors and that women and unemployed individuals were less likely to report such increases. We also found that individuals with anxiety were more likely to report increases in addictive behaviors than individuals who did not have anxiety. Together, these findings provide important insights regarding who may be at increased risk for adopting unhealthy behaviors and could potentially benefit from lifestyle interventions.

Consistent with previous research [35,36], we found that greater adherence to social distancing guidelines was associated with self-reported improvements in healthy lifestyle behaviors. Individuals who adhered more to social distancing guidelines may have experienced an increase in leisure time and used that time to prepare healthy meals and stay physically active. Meanwhile, women were less likely to report increases in healthy lifestyle behaviors. This finding may reflect the larger societal strain and burden experienced by women during the COVID-19 pandemic [37], due in part to the closure of schools and day care centers [38]. In addition, unemployed individuals were less likely to report increases in healthy lifestyle behaviors. Being unemployed may lead to greater dependency on relatively cheaper (and unhealthy) fast foods [39], and previous research in U.S. adults has found that unemployment was associated with reductions in daily PA [40]. Overall, our findings suggest that women and unemployed individuals are at increased risk for weight gain and sedentary lifestyle during the pandemic. As such, they may benefit from interventions that emphasize healthy eating and PA and teach problem-solving and coping skills to address the additional stressors brought on by the pandemic that may be contributing to decrements in a healthy lifestyle.

Results of the multivariable analysis revealed that individuals with anxiety were more likely to report increases in addictive behaviors. Although this finding is consistent with other pandemic-focused studies [18,41], it is notable because almost half of our survey respondents scored above the PROMIS threshold and had significant anxiety symptoms. Put into context, estimates from the National Health Interview Survey from January to June 2019 showed that 8.2% of the U.S. adult population had symptoms of anxiety disorder [42], suggesting significantly elevated levels of anxiety during the COVID-19 pandemic. The substantial rise in anxiety and corresponding increase in unhealthy lifestyle behaviors may portend future behavioral and health consequences. As tobacco and alcohol are addictive substances, smoking and drinking could become the norm for substantial numbers of U.S. adults who are trying to combat pandemic-induced anxiety. Indeed, some have already called for more public health warnings about excessive substance use during this unprecedented time [43]. Although more research is needed to understand the longitudinal associations between pandemic-induced anxiety and unhealthy lifestyle behaviors, our findings suggest that individuals with high anxiety levels may be at increased risk for developing substance use problems and could potentially benefit from smoking cessation and substance use prevention interventions.

This study had some limitations. First, it was based on an online survey which excludes the possibility of verifying the data on objective grounds. However, considering the challenges of conducting such a study during pandemic lockdown, this limitation was impossible to overcome. Moreover, there is evidence that web-based surveys are equivalent to conventional face-to-face interviews in terms of data quality [44,45]. Second, given the cross-sectional nature of the data, findings represent a snapshot of lifestyle behaviors at a single moment in time. We are unable to account for how behaviors may evolve over time. Third, the descriptive and analytic inferences made are generalizable to the U.S. adult population under the assumption that non-response is unrelated to any of the sociodemographic factors examined.

This study also had some notable strengths. First, it is one of the largest studies to date to examine individual-level determinants of healthy and addictive lifestyle behavioral changes in response to the COVID-19 pandemic. Second, our study sample was racially, ethnically, socioeconomically, and geographically diverse, which increases generalizability. Third, most studies examining lifestyle changes during the COVID-19 pandemic have either focused on the magnitude of change or sought to examine the effects of a single class of determinants (e.g., mental health/well-being [4,46] or sociodemographic factors [47]) on behavioral change. This approach fails to consider the effect of other individual-level variables. Our multivariable analytic approach addresses this knowledge gap by controlling for multiple individual-level determinants of lifestyle behavioral change (i.e., sociodemographic, mental health, and behavioral).

## 5. Conclusions

This study provides new data on lifestyle behavioral changes during the COVID-19 pandemic lockdown in the general U.S. population. Overall, findings suggest that women, unemployed individuals, and those with high anxiety levels are at increased risk of unhealthy lifestyle behavioral changes during the COVID-19 pandemic, but that they may benefit from differently focused lifestyle interventions. Whereas women and unemployed individuals may benefit from lifestyle interventions targeting diet and physical activity, individuals with anxiety may benefit from lifestyle interventions targeting smoking and alcohol cessation. Since the COVID-19 pandemic is still ongoing, more extensive population studies of lifestyle behavioral changes are warranted to confirm our results and understand the long-term effects of the current crisis on physical health.

## Figures and Tables

**Table 1:** Descriptive analysis of participants’ characteristics (*n* = 1276).

Sociodemographic Characteristics	*n* (%) ^a^	Lifestyle Behaviors and Self-Reported Changes Since the Pandemic	*n* (%) ^a^
Age, Mean (SD), years	45.0 (17.0)	Drinks alcohol	
18–30	324 (25.4)	Yes	562 (58.2)
31–50	489 (38.3)	No	404 (41.8)
51–65	264 (20.7)	Reported change in alcohol use	
>65	199 (15.6)	Increased	218 (39.5)
Gender		Decreased	87 (15.8)
Male	724 (57.5)	Stayed the same	247 (44.8)
Female	517 (41.0)	Vaping frequency	
Race/Ethnicity		Daily	108 (11.3)
White	623 (50.2)	Less than Daily	64 (6.7)
Black	238 (19.2)	Not at all	788 (82.0)
Hispanic	181 (14.6)	Reported change in vaping frequency	
Asian	35 (2.8)	Increased	78 (45.9)
Other	165 (13.3)	Decreased	31 (18.2)
Education		Stayed the same	61 (35.9)
Not college educated	263 (21.0)	Tobacco smoking frequency	
College educated	988 (79.0)	Daily	181 (19.0)
Marital status		Less than daily	61 (6.4)
Unmarried	613 (48.8)	Not at all	713 (74.7)
Married	644 (51.2)	Reported change in tobacco smoking frequency	
Annual household income		Increased	98 (41.0)
Less than $25 K	185 (19.0)	Decreased	48 (20.1)
$25 K to $74 K	382 (39.3)	Stayed the same	93 (38.9)
$75 K or more	406 (41.7)	Increase in PA frequency	
Number of household residents		1 = Strongly Disagree	718 (18.6)
1	251 (20.1)	2 = Disagree	224 (23.4)
2	408 (32.6)	3 = Neutral	237 (24.8)
3–4	433 (34.6)	4 = Agree	205 (21.4)
5 or more	158 (12.6)	5 = Strongly Agree	113 (11.8)
Lives with someone > age 65		Increase in healthy eating	
Yes	281 (28.1)	1 = Strongly disagree	75 (7.8)
No	720 (71.9)	2 = Disagree	213 (22.3)
Lives with someone < age 18		3 = Neutral	324 (33.9)
Yes	452 (45.3)	4 = Agree	243 (25.4)
No	546 (54.7)	5 = Strongly agree	102 (10.7)
Work status		Mental Health	
Working full-time	460 (47.2)	Anxiety ^b^	
Working part-time	128 (13.1)	T-score, mean (SE)	58.9 (10.6)
Retired	165 (16.9)	Case	423 (47.7)
Unemployed	221 (22.7)	Not a case	464 (52.3)
Living arrangement		Depression ^b^	
Lives alone	229 (18.6)	T-score, mean (SE)	56.1 (10.1)
Lives with spouse/partner	679 (55.3)	Case	347 (39.1)
Lives with a family member	274 (22.3)	Not a case	541 (60.9)
Lives with a non-family member	47 (3.8)		
US region of residence		COVID-19 mitigation behaviors	
Northeast	205 (21.5)	Area of residence under stay-at-home order	
Midwest	200 (21.0)	Yes	803 (82.8)
South	365 (38.3)	No	143 (14.7)
West	184 (19.3)	Stay-at-home adherence ^c^	
		Continuing life as normal	10 (1.3)
		Stay at home besides essential trips	269 (33.5)
		Social distancing adherence ^d^	
		No social distancing	14 (1.5)
		Complete social distancing	299 (31.9)
		Hand hygiene/sanitization adherence ^e^	
		Never	12 (1.3)
		Every few hours	327 (34.9)

^a^ “Missing” was shown for reference. Missing data was not included in statistical analyses. ^b^ Individuals categorized as having depression or anxiety met the criteria for “caseness” (T-score > 60) on the PROMIS 4-item short-form depression and anxiety measures. ^c^ “Continuing life as normal” is 0 on 0 to 10 scale, “stay at home besides essential trips” is 10 on 0 to 10 scale. ^d^ “No social distancing” is 0 on 0 to 10 scale, “complete social distancing” is 10 on 0 to 10 scale. ^e^ “never” is 0 on 0 to 10 scale, “every few hours” is 10 on 0 to 10 scale.

**Table 2:** Univariate and multivariable linear regression analyses showing healthy behavioral change. Index ^a^ as a function of individual-level determinants ^b^.

Factors	Healthy Behavioral Change Index ^a^					
	Crude Regression Coefficients	95% CI	*p*-Value	Adjusted Regression Coefficients ^b^	95% CI	*p*-Value
**Age**						
One unit increase	−0.11	−0.02, −0.01	<0.001			
18–30	Ref		<0.001	Ref		0.63
31–50	0.10	−0.13, 0.33		−0.04	−0.34, 0.27	
51–65	−0.36	−0.62, −0.11		−0.23	−0.60, 0.15	
>65	−0.40	−0.68, −0.12		−0.10	−0.64, 0.44	
**Gender**			<0.001			
Male	Ref			Ref		
Female	−0.90	−0.57, −0.22		−0.37	−0.62, −0.12	0.003
**Race**			<0.001			0.35
White	Ref			Ref		
Black	0.54	0.31, 0.76		0.32	−0.02, 0.66	
Hispanic	0.33	0.06, 0.60		0.26	−0.09, 0.61	
Asian	0.45	−0.02, 0.93		0.21	−0.43, 0.85	
Other	0.27	−0.04, 0.59		0.07	−0.31, 0.45	
**Education**			0.02			0.12
Not college educated	Ref			Ref		
College educated	0.27	0.05, 0.49		0.24	−0.06, 0.55	
**Marital status**			0.006			0.34
Unmarried	Ref			Ref		
Married	0.24	0.07, 0.42		0.13	−0.13, 0.39	
**Annual household income**			0.002			0.24
Less than $25,000	Ref			Ref		
$25,000 to $74,000	0.14	−0.11, 0.38		−0.18	−0.53, 0.18	
$75,000 or more	0.41	0.16, 0.65		0.03	−0.34, 0.40	
**Living arrangement**			0.08			
Lives alone	Ref					
Lives with spouse/partner	0.27	0.03, 0.50				
Lives with family member	0.22	−0.05, 0.50				
Lives with non-family member	−0.07	−0.53, 0.38				
**Number of household residents**			0.22			
1	Ref					
2	0.10	−0.14, 0.35				
3–4	0.22	−0.02, 0.47				
5 or more	0.26	−0.06, 0.59				
**Lives with someone > age 65**			0.01			0.75
Yes	−0.29	−0.51, −0.06		0.05	−0.27, 0.37	
No	Ref			Ref		
**Lives with child < age 18**			<0.001			0.10
Yes	0.41	0.21, 0.60		0.22	−0.04, 0.47	
No	Ref			Ref		
**Work status**			<0.001			
Working full-time	Ref					0.10
Working part-time	−0.25	−0.51, −0.02		−0.18	−0.52, 0.15	
Retired	−0.71	−0.95, −0.47		−0.43	−0.88, 0.02	
Unemployed	−0.58	−0.79, −0.36		−0.33	−0.64, −0.02	
**Anxiety** ^c^						
Case	−0.03	−0.21, 0.15				
Not a case	Ref					
**Depression** ^c^			0.30			
Case	−0.10	−0.28, 0.09				
Not a case	Ref					
**Stay-at-home adherence**			0.01			0.77
One unit increase	0.06	0.01, 0.11		0.01	−0.06, 0.09	
**Social distancing adherence**			<0.001			0.004
One unit increase	0.09	0.04, 0.13		0.12	0.04, 0.21	
**Hand hygiene/sanitization adherence**						0.79
One unit increase	0.07	0.03, 0.11	0.001	0.01	−0.04, 0.06	

^a^ The healthy behavioral change index includes physical activity and healthy eating, and scores range from +2 to −2. ^b^ Individual-level determinants include sociodemographic, mental health, and behavioral (i.e., adherence to COVID-19 mitigation strategies) factors. ^c^ Living arrangement was not included in the final model due to collinearity with lives with someone aged >65 and lives with child aged <18. ^c^ Individuals categorized as having depression or anxiety met the criteria for “caseness” (T-score > 60) on the PROMIS 4-item short-form depression and anxiety measures.

**Table 3:** Univariate and multivariable linear regression analyses showing addictive behavior. Change index ^a^ as a function of individual-level determinants ^b^.

Factors	Addictive Behavioral Change Index ^a^					
	Crude Regression Coefficients	95% CI	*p*-Value	Adjusted Regression Coefficients ^c^	95% CI	*p*-Value
**Age**						
One unit increase	−0.005	−0.008, −0.001	0.005			
18–30	Ref		0.002			0.98
31–50	0.07	−0.08, 0.23		−0.003	−0.19, 0.19	
51–65	−0.08	−0.25, 0.09		−0.04	−0.26, 0.18	
>65	−0.19	−0.38, −0.01		0.01	−0.27, 0.30	
**Gender**			0.68			
Male	Ref					
Female	−0.024	−0.14, 0.09				
**Race**						
White	Ref					
Black	0.13	−0.03, 0.28				
Hispanic	−0.09	−0.28, 0.09				
Asian	−0.19	−0.51, 0.13				
Other	0.09	−0.12, 0.30				
**Education**			0.43			
Not college educated	Ref					
College Educated	0.06	−0.09, 0.21				
**Marital status**			0.59			
Unmarried	Ref					
Married	0.03	−0.08, 0.15				
**Annual household income**			0.74			
Less than $25,000	Ref					
$25,000 to $74,999	0.06	−0.10, 0.23				
$75,000 or more	0.04	−0.13, 0.20				
**Living arrangement**			0.04			
Lives alone	Ref					
Lives with spouse/partner	0.17	0.01, 0.32				
Lives with family member	0.11	−0.07, 0.29				
Lives with non-family member	0.40	0.10, 0.71				
**Number of household residents**			0.008			0.5
1	Ref			Ref		
2	0.08	−0.08, 0.24		−0.03	−0.53, 0.48	
3–4	0.26	0.10, 0.42		0.02	−0.48, 0.53	
5 or more	0.13	−0.08, 0.35		−0.14	−0.67, 0.39	
**Lives with someone > age 65**			0.28			
Yes	−0.08	−0.23, 0.06				
No	Ref					
**Lives with child < age 18**			0.004			0.12
Yes	0.19	0.06, 0.32		0.14	−0.04, 0.32	
No	Ref			Ref		
**Work status**						0.38
Working full-time	Ref		0.01	Ref		
Working part-time	−0.01	−0.19, 0.17		−0.03	−0.24, 0.18	
Retired	−0.27	−0.43, −0.10		−0.20	−0.47, 0.07	
Unemployed	−0.11	−0.26, 0.04		−0.11	−0.29, 0.06	
**Anxiety** ^d^			<0.0001			0.002
Case	0.35	0.23, 0.47		0.26	0.09, 0.43	
Not a case	Ref			Ref		
**Depression** ^d^			<0.0001			0.36
Case	0.28	0.16, 0.40		0.08	−0.09, 0.25	
Not a case	Ref			Ref		
**Stay-at-home adherence**						
One unit increase	−0.002	−0.037, 0.033	0.90			
**Social distancing adherence**						
One unit increase	0.02	−0.01, 0.05	0.19			
**Hand hygiene/sanitization adherence**						0.47
One unit increase	0.02	−0.16, 0.27	0.09	0.01	−0.02, 0.04	

^a^ The addictive behavioral change index includes alcohol use, tobacco smoking, and vaping. Scores range from +3 to −3. ^b^ Individual-level determinants include sociodemographic, mental health, and behavioral (i.e., adherence to COVID-19 mitigation strategies) factors. ^c^ Living arrangement was not included into final model due to collinearity with number of household residents and lives with child < age 18. ^d^ Individuals categorized as having depression or anxiety met the criteria for “caseness” (T-score > 60) on the PROMIS 4-item short-form depression and anxiety measures.

## Data Availability

The datasets generated and/or analyzed during the current study are not publicly available but are available from the corresponding author on reasonable request.

## Supplementary Materials

The following are available online at https://www.mdpi.com/article/10.3390/ijerph18084364/s1, Table S1: Associations between individual level determinants and healthy behavioral change (physical activity and healthy eating), Table S2: Associations between individual level determinants and addictive behavioral change (tobacco smoking, alcohol use and vaping).

## Appendix A

**Table A1:** Survey measures.

Construct	Measure
Sociodemographics (19 items)	Age, gender, race/ethnicity, education, marital status, annual household income, work status, current living arrangement (alone, or with a spouse/partner, family member, or non-family member), number of household residents, whether they lived with someone over age 65 or younger than age 18, zip code, and cross street.
**Mental Health Impacts**	
General Depression (4 items)	PROMIS Depression 4-item Short form [32]
General Anxiety (4 items)	PROMIS Anxiety 4-item Short Form [33]
**Health Behavioral Impacts: COVID-19 Preventive Measures**	
Adherence to Stay-at-Home Orders	“Does the area where you live have a stay-at-home Orders?” (1 = yes, 2 = no, 3 = I don’t know) “To what extent do you currently follow the stay at home order?” (0 = not at all to 10 = completely)
Social Distancing	“What amount of social distancing do you currently practice?” (0 = no social distancing to 10 = complete social distancing)
Hand Hygiene	“How often do you practice protective measures like hand washing, use of hand sanitizer, or disinfection of household surfaces to keep yourself and others you live with from contracting COVID-19?” (0 = never to 10 = every few hours)
**Health Behavioral Impacts: Lifestyle Behaviors**	
Alcohol Use	“Has your drinking increased/decreased/stayed the same since COVID-19?”
Tobacco Use	Two items on current smoking status and type and number of tobacco products smoked per day, taken from the Global Adult Tobacco Survey [31]
Exercise	“Since COVID-19 I am exercising more.” (1 = strongly disagree to 5 = strongly agree)
Diet	“Since COVID-19 I am eating more healthy foods.” (1 = strongly disagree to 5 = strongly agree)
