# Supplementary material for: Individual-Level Determinants of Lifestyle Behavioral Changes during COVID-19 Lockdown in the United States: Results of an Online Survey
Source: Int J Environ Res Public Health. 2021 Apr 20;18(8):4364. doi: 10.3390/ijerph18084364 (PMC8073729; doi:10.3390/ijerph18084364)
Supplement: Supplementary file 1 [file ijerph-18-04364-s001.zip › ijerph-1175344-SI.pdf]

Table S1. Associations between individual level determinants and healthy behavior change (physical activity and healthy eating)

		Physical activity				Healthy eating			
		**PA decreased**	**PA increased**	**Stay the same**	**p-value**	**Eating less healthy**	**Eating more healthy**	**Stay the same**	**p-value**
**Age**	Mean (SD)	48.6 (16.60	42.4 (15.8)	47.2 (17.5)	0.23	46.1 (16.3)	43.9 (16.1)	48.9 (17.5)	0.15
**Gender**					<0.01				0.21
	Male	180 (40.3)	147 (32.9)	120 (26.9)		126 (28.3)	174 (39.0)	146 (32.7)	
	Female	134 (27.3)	248 (50.5)	109 (22.2)		156 (31.7)	165 (33.5)	171 (34.8)	
**Race**					<0.01				0.18
	White	144 (27.4)	254 (48.3)	128 (24.3)		174 (33.1)	172 (32.7)	180 (34.2)	
	Black	89 (46.8)	54 (28.4)	47 (24.7)		49 (25.9)	74 (39.2)	66 (34.9)	
	Hispanic	43 (36.1)	43 (36.1)	33 (26.5)		32 (26.7)	46 (38.3)	42 (35.0)	
	Asian	12 (35.3)	13 (38.2)	9 (26.5)		6 (18.2)	16 (48.5)	11 (33.3)	
	Other	29 (34.9)	35 (42.2)	19 (22.9)		25 (29.8)	37 (44.1)	22 (26.2)	
**Education**					<0.01				0.10
	Not College Educated	43 (24.3)	77 (43.5)	57 (32.2)		63 (35.4)	53 (29.8)	62 (34.8)	
	College Educated	275 (35.3)	325 (41.7)	179 (23.0)		225 (28.9)	292 (37.5)	261 (33.6)	
**Marital status**					0.03				0.02
	Unmarried	136 (29.2)	206 (44.2)	124 (26.6)		148 (31.9)	146 (31.5)	170 (36.6)	
	Married	182 (37.1)	195 (39.8)	113 (23.1)		140 (28.5)	198 (40.2)	154 (31.3)	
**Annual Household Income**					<0.01				0.49
	Less than $25K	40 (22.6)	89 (50.3)	48 (27.1)		55 (30.9)	58 (32.60	65 (36.5)	
	$25K to $74K	104 (28.7)	157 (73.4)	101 (27.9)		114 (31.6)	124 (34.4)	123 (34.1)	
	$75K or more	163 (42.2)	148 (38.3)	75 (19.4)		110 (28.5)	152 (39.4)	124 (32.1)	

**Living arrangement**					0.22				0.01
	Living alone	50 (27.9)	78 (43.6)	51 (28.5)		58 (32.6)	49 (27.5)	71 (39.9)	
	Living with spouse/romantic partner	185 (36.1)	209 (40.8)	118 (23.1)		148 (28.9)	199 (38.8)	166 (32.4)	
	Living with a family member	60 (29.3)	87 (42.4)	58 (28.3)		53 (25.9)	83 (40.5)	69 (33.7)	
	Living with a non-family member	16 (38.1)	19 (45.2)	7 (16.7)		2 (47.6)	11 (26.2)	11 (26.2)	
**Number of household residents**					0.34				0.03
	1	59 (30.4)	82 (42.3)	53 (27.3)		63 (32.5)	55 (28.4)	76 (39.2)	
	2	94 (30.2)	141 (45.3)	76 (24.4)		85 (27.2)	111 (35.6)	116 (37.2)	
	3~4	129 (38.2)	132 (39.1)	77 (22.8)		109 (32.3)	129 (38.3)	99 (29.4)	
	5 or more	35 (32.1)	44 (40.4)	30 (27.5)		30 (27.5)	49 (45.0)	30 (27.5)	
**Lives with someone > age 65**					0.055				0.07
	Yes	59 (28.5)	98 (47.3)	50 (24.2)		64 (30.8)	67 (32.2)	77 (37.0)	
	No	205 (37.2)	215 (39.0)	131 (23.8)		158 (28.7)	226 (41.0)	167 (30.3)	
**Lives with child < age 18**					<0.01				<0.01
	Yes	136 (41.2)	111 (33.6)	83 (25.2)		90 (27.3)	148 (44.9)	92 (27.9)	
	No	124 (29.1)	202 (47.4)	100 (23.5)		131 (30.7)	143 (33.5)	153 (35.8)	
**Work status**					<0.01				<0.01
	Working full time	192 (42.8)	149(33.2)	108 (24.1)		127 (28.2)	200 (44.4)	124 (27.5)	
	Working part time	44 (34.9)	51 (40.5)	31 (24.6)		38 (30.4)	45 (36.0)	42 (33.6)	
	Retired	28 (17.5)	87 (54.4)	45 (28.1)		51 (31.9)	37 (23.1)	72 (45.0)	
	Unemployed	53 (24.4)	112 (51.6)	52 (24.0)		71 (32.9)	62 (28.7)	83 (38.4)	
**Anxiety**					0.49				0.6209
	Mean (SD)	59.6 (10.9)	58.8 (10.5)	59.6 (10.9)		59.7 (10.8)	59.4 (10.8)	57.7 (10.3)	

	Yes	141 (33.3)	185 (43.7)	97 (22.9)		127 (30.1)	158 (37.4)	137 (32.5)	
	No	158 (34.1)	186 (40.2)	119 (25.7)		137 (29.6)	162 (35.0)	164 (35.4)	
**Depression**					0.85				0.0578
	Mean (SD)	56.5 (10.1)	55.8 (10.4)	56.5 (10.1)		57.1 (10.4)	56.4 (10.1)	54.9 (9.7)	
	Yes	112 (32.4)	149 (43.1)	85 (24.6)		118 (34.0)	125 (36.0)	104 (30.0)	
	No	185 (34.2)	226 (41.8)	130 (24.0)		148 (27.5)	194 (36.0)	197 (36.6)	
**Stay at home adherence**									
	Mean (SD)	8.4 (2.0)	8.5 (1.7)	8.3 (2.0)	0.76	8.1 (2.1)	8.7 (1.7)	8.4 (1.80	0.92
**Social distancing adherence**									
	Mean (SD)	8.2 (2.1)	8.5 (1.7)	8.0 (2.2)	0.06	7.8 (2.3)	8.6 (1.7)	8.2 (2.0)	0.49
**Hand hygiene/sanitization adherence**									
	Mean (SD)	8.2 (2.0)	7.8 (2.4)	7.9 (2.2)	0.41	7.7 (2.4)	8.3 (2.0)	7.9 (2.3)	0.13

Table S2. Associations between individual level determinants and addictive behavior change (tobacco smoking, alcohol use and vaping)

		**Tobacco Smoking**				**Alcohol Consumption**				**Vaping**			
		**Smoking increased**	**Smoking decreased**	**Stay the same**	**p-value**	**Alcohol drinking increased**	**Alcohol drinking decreased**	**Stay the same**	**p-value**	**Vaping increased**	**Vaping decreased**	**Stay the same**	**p-value**
**Age**	Mean (SD)	41.8 (14.8)	40.2 (12.6)	46.4 (15.3)	0.64	41.4 (13.1)	41.0 (15.4)	50.7 (17.3)	0.85	35.2 (10.4)	38.4 (12.1)	38.3 (12.1)	0.30
**Gender**					0.12				0.054				0.74
	Male	59 (37.1)	37 (23.3)	63 (39.6)		115 (39.4)	56 (19.2)	121 (41.4)		52 (45.6)	23 (20.2)	39 (34.2)	
	Female	36 (49.3)	10 (13.7)	27 (37.0)		97 (38.5)	31 (12.3)	124 (49.2)		20 (42.6)	8 (17.0)	19 (40.4)	
**Race**					0.46				0.17				0.58
	White	40 (37.7)	21 (19.8)	45 (42.5)		115 (38.5)	39 (13.0)	145 (48.5)		30 (48.4)	8 (12.9)	24 (38.7)	
	Black	37 (42.5)	16 (18.4)	34 (39.1)		59 (45.4)	21 (16.2)	50 (38.5)		24 (47.1)	13 (25.5)	14 (27.5)	
	Hispanic	8 (36.4)	8 (36.4)	6 (27.3)		19 (31.7)	16 (26.7)	25 (41.7)		17 (43.6)	8 (20.5)	14 (35.9)	
	Asian	1 (33.3)	1 (33.3)	1 (33.3)		4 (28.6)	3 (21.4)	7 (50.0)		1 (25.0)	1 (25.0)	2 (50.0)	
	Other	12 (57.1)	2 (9.5)	7 (33.3)		21 (43.8)	8 (16.7)	19 (39.6)		6 (42.9)	1 (7.1)	7 (50.0)	
**Education**					0.90				0.20				0.20
	Not College Educated	27 (42.9)	13 (20.6)	23 (36.5)		30 (34.9)	19 (22.1)	37 (43.0)		20 (40.8)	13 (26.5)	16 (32.7)	
	College Educated	71 (40.3)	35 (19.9)	70 (39.8)		188 (40.3)	68 (14.6)	210 (45.1)		58 (47.9)	18 (14.9)	45 (37.2)	
**Marital status**					0.02				0.02				0.26
	Unmarried	57 (43.5)	18 (13.7)	56 (42.8)		101 (39.6)	51 (20.0)	103 (40.4)		32 (40.0)	15 (18.8)	33 (41.3)	
	Married	41 (38.3)	30 (28.0)	36 (33.6)		117 (39.4)	36 (12.1)	144 (48.5)		46 (51.7)	16 (18.0)	27 (30.3)	
**Annual Household Income**					0.39				0.66				0.996
	Less than $25K	22 (46.8)	8 (17.0)	17 (36.2)		24 (35.3)	13 (19.1)	31 (45.6)		12 (48.0)	4 (16.0)	9 (36.0)	
	$25K to $74K	39 (41.5)	14 (14.9)	41 (43.6)		81 (39.1)	28 (13.5)	98 (47.3)		32 (47.1)	13 (19.1)	23 (33.8)	
	$75K or more	36 (39.6)	23 (25.3)	32 (35.2)		107 (41.2)	43 (16.5)	110 (42.3)		33 (45.8)	13 (18.1)	26 (36.1)	

**Living arrangement**				0.74				0.02				0.94
Living alone	15 (33.3)	10 (22.2)	20 (44.4)		27 (30.7)	18 (20.5)	43 (48.9)		13 (43.3)	8 (26.7)	9 (30.0)	
Living with spouse/romantic partner	51 (42.5)	23 (19.2)	46 (38.3)		125 (38.5)	41 (12.6)	159 (48.9)		40 (45.5)	15 (17.1)	33 (37.50	
Living with a family member	19 (35.9)	11 (20.8)	23 (43.4)		44 (44.4)	22 (22.2)	33 (33.3)		17 (46.0)	6 (16.2)	14 (37.8)	
Living with a non-family member	9 (56.3)	3 (18.8)	4 (25.0)		13 (52.0)	4 (16.0)	8 (32.0)		4 (40.0)	2 (20.0)	4 (40.0)	
**Number of household residents**				0.32				0.01				0.62
1	17 (34.7)	11 (22.9)	21 (22.8)		31 (31.3)	21 (21.2)	47 (47.5)		13 (41.9)	8 (25.8)	10 (32.2)	
2	30 (39.0)	12 (25.0)	35 (38.0)		61 (33.9)	28 (15.6)	91 (50.6)		14 (35.0)	8 (20.0)	18 (45.0)	
3~4	39 (42.4)	23 (47.9)	30 (32.6)		102 (47.9)	25 (11.7)	86 (40.4)		40 (51.3)	12 (15.4)	26 (33.3)	
5 or more	11 (57.9)	2 (10.5)	6 (31.6)		23 (40.4)	12 (22.8)	21 (36.8)		10 (50.0)	3 (15.0)	7 (35.0)	
**Lives with someone > age 65**				0.76				<0.01				0.53
Yes	23 (46.0)	9 (18.0)	18 (36.0)		32 (27.8)	12 (10.4)	71 (61.7)		13 (48.2)	6 (22.2)	8 (29.6)	
No	56 (40.0)	29 (20.7)	55 (39.3)		151 (44.9)	56 (16.7)	129 (38.4)		49 (44.6)	17 (15.5)	44 (40.0)	
**Lives with child < age 18**				0.41				<0.01				0.51
Yes	41 (44.6)	20 (21.7)	31 (33.7)		100 (50.5)	27 (13.6)	71 (35.9)		41 (48.2)	15 (17.7)	29 (34.1)	
No	37 (39.0)	17 (17.9)	41 (43.2)		83 (32.8)	41 (16.2)	129 (51.0)		21 (42.0)	7 (14.0)	22 (44.0)	
**Work status**				0.33				<0.01				0.29
Working full time	57 (41.9)	32 (23.5)	47 (34.6)		139 (44.7)	55 (17.7)	117 (37.6)		53 (44.5)	21 (17.7)	45 (37.8)	
Working part time	14 (48.3)	5 (17.2)	10 (34.5)		27 (43.6)	8 (12.9)	27 (43.6)		13 (61.9)	4 (19.1)	4 (19.1)	
Retired	6 (27.3)	3 (13.6)	13 (59.1)		11 (14.7)	7 (9.3)	57 (76.0)		0 (0.0)	1 (25.0)	3 (75.0)	
Unemployed	20 (39.2)	8 (15.7)	23 (45.1)		41 (40.2)	17 (16.7)	44 (43.1)		12 (46.2)	5 (19.2)	9 (34.6)	
**Anxiety**				<0.01				<0.01				0.03

	Mean (SD)	65.6 (8.8)	60.4 (9.4)	56.6 (11.2)		62.7 (9.2)	59.8 (10.6)	57.4 (9.9)		66.4 (8.7)	61.1 (9.3)	62.1 (9.9)	
	Yes	67 (52.3)	22 (17.2)	39 (30.5)		133 (51.6)	37 (14.3)	88 (34.1)		57 (52.8)	17 (15.7)	34 (31.5)	
	No	24 (25.8)	22 (23.7)	47 (50.5)		70 (27.9)	40 (15.9)	141 (56.2)		15 (30.6)	13 (26.5)	21 (42.9)	
**Depression**					<0.01				<0.01				0.04
	Mean (SD)	63.4 (8.6)	58.5 (8.0)	55.4 (10.1)		59.8 (9.6)	56.7 (10.1)	53.5 (9.9)		63.3 (8.8)	60.0 (7.9)	60.2 (9.4)	
	Yes	59 (53.2)	20 (18.0)	32 (28.8)		105 (50.2)	33 (15.8)	71 (34.0)		50 (54.4)	14 (15.2)	28 (30.4)	
	No	32 (29.1)	23 (20.9)	55 (50.0)		100 (33.0)	42 (13.9)	161 (53.1)		22 (33.9)	16 (24.6)	27 (41.5)	
**Stay at home adherence**													
	Mean (SD)	8.4 (2.0)	8.3 (2.0)	8.6 (1.4)	0.97	8.3 (2.1)	8.6 (1.3)	8.4 (1.7)	0.63	8.7 (1.5)	8.5 (1.6)	8.3 (2.1)	0.73
**Social distancing adherence**													
	Mean (SD)	7.8 (2.2)	7.8 (2.2)	7.9 (2.2)	0.43	8.3 (2.0)	8.2 (1.7)	8.1 (2.0)	0.03	8.2 (2.1)	7.9 (2.0)	7.9 (2.2)	0.14
**Hand hygiene/sanitization adherence**													
	Mean (SD)	8.3 (1.9)	7.8 (2.1)	7.8 (2.2)	0.78	8.0 (2.2)	7.8 (2.1)	7.9 (2.3)	0.82	8.3 (1.8)	7.3 (2.3)	8.2 (2.1)	0.46
